# Rudy and Borden technique for penile self-mutilation in Klingsor syndrome: a case report

**DOI:** 10.11604/pamj.2021.38.334.28897

**Published:** 2021-04-07

**Authors:** Imad Boualaoui, Hicham El Bote, Omar Bellouki, Ahmed Ibrahimi, Hachem El Sayegh, Yassine Nouini

**Affiliations:** 1 Department of Urology A, Mohammed V University in Rabat, Ibn Sina University Hospital, Rabat, Morocco

**Keywords:** Genitalia, self-mutilation, surgical flaps, psychotic disorders, case report

## Abstract

Penile self-mutilation is a challenging situation that often jeopardizes sexual and voiding functions. Surgical treatment is currently based on penile replantation, nevertheless its requirements of time, conservation, and quality of amputated phallus and microsurgical expertise are not constantly available. Here, we present a case of penile self-inflicted amputation in a 28-years-old patient suffering from a psychotic disorder, who did not preserve the amputated phallus. In the first clinical examination, we have attested a hemorrhagic total penile section, 6cm from the penoscrotal angle. We performed immediate surgical management. Rudy and Borden technique is a reconstructive surgery procedure with interesting functional results, by performing a dorsal vascularized split-thickness skin flap to cover the penile shaft. The aim of this technique is to avoid perineostomy which compounds significantly the quality of life.

## Introduction

Genitalia self-mutilation (GSM) is a dramatic event that is frequently but not merely occurs within severe psychiatric disorders [1]. Penile self-mutilation is a challenging situation that often jeopardizes sexual and voiding functions, furthermore social and especially psychological aspects of self-inflicted penile amputation intricate frequently the overall management [2]. Surgical treatment is currently based on penile replantation, nevertheless its requirements of time, conservation, and quality of amputated phallus and microsurgical expertise are not constantly available. Rudy and Borden technique is an organ-sparing reconstructive surgery procedure, first described in localized glan penis tumors after a partial or total glansectomy [3,4]. Herein, we report a case of a patient with a history of recurrent intentional male genitalia self-mutilation and discuss surgical and psychological issues we faced.

## Patient and observation

**Figure 1:**
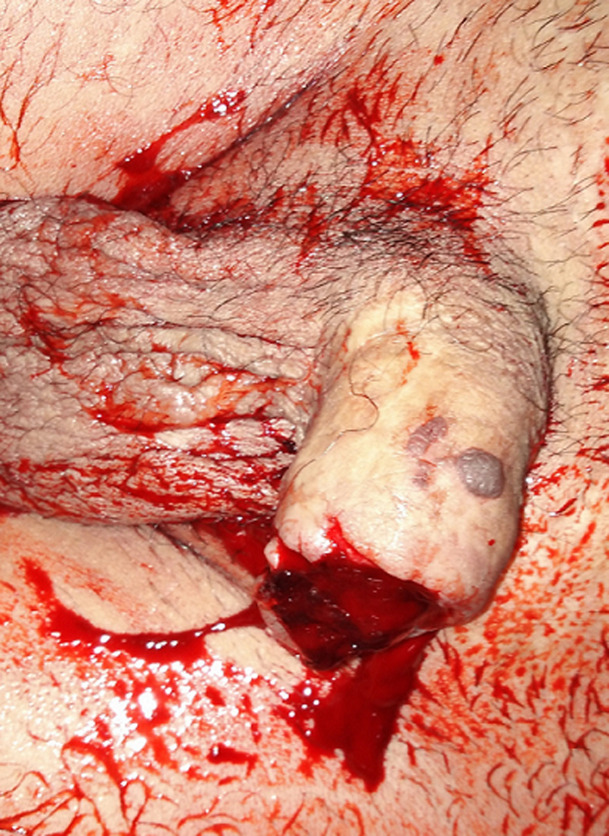
hemorrhagic total penile section, 6cm from the penoscrotal angle

**Figure 2:**
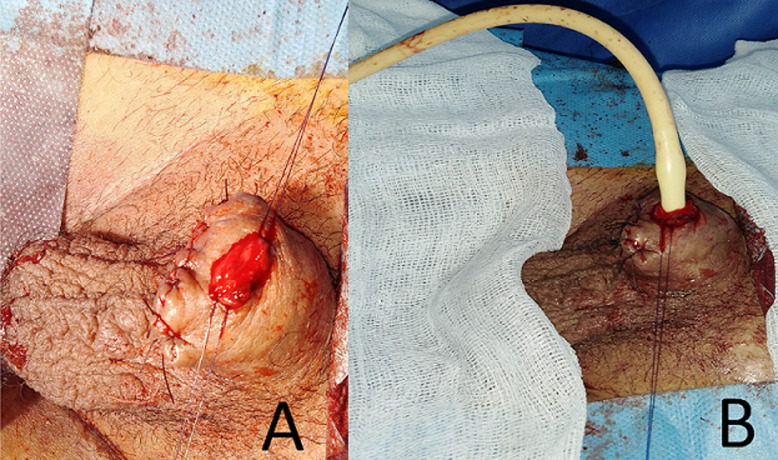
A) a dorsal vascularized split-thickness skin flap covered the penile shaft; B) the urethrocutaneous suture was done by a 5-0 polydioxanon thread, and the urethral catheter was kept for two weeks

**Figure 3:**
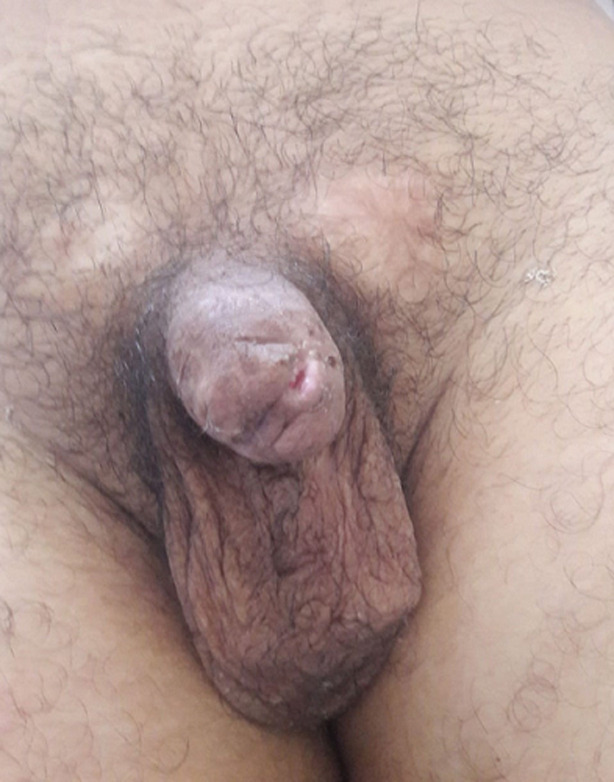
the result after a year of follow-up

A 28-years-old patient came with his mother to our emergency unit after a penile self-mutilation. He suffered from a psychiatric illness with a psychotic disorder characterized by a schizophrenia paranoid type. His genitalia self-mutilation history began two years ago by a self-inflicted castration. In the first clinical examination, we have attested a hemorrhagic total penile section, 6cm from the penoscrotal angle (Figure 1). The patient did not conserve the sliced penile fragment. The scrotal cavity was empty, and there was no other self-mutilation elsewhere. He was calm, but soft and apathetic. His mother confirmed a complete therapeutic inobservance. We concluded to a klingsor syndrome. We performed immediate surgical management. The first aim of emergency surgery was to stop bleeding and then to preserve the compromised urinary and sexual function. We did not perform any imaging test and decided to follow Rudy and Borden technique. After performing general anesthesia and placing a base penile tourniquet, the surgical procedure began with wound debridement and massive flushing by sterile saline. We transversely aligned the cavernous section slice and ligated the injured largest dorsal penile vein. Afterward, we closed the corporeal bodies albuginea with 4-0 polydioxanone suture and kept 1cm of urethra downstream which was spatulate in 5mm. A dorsal vascularized split-thickness skin flap covered the penile shaft. We prepared the neomeatus by performing a three-cornered opening in the flap skin. The urethrocutaneous suture was done by a 5-0 polydioxanone thread (Figure 2). We kept the urethral catheter for two weeks. After a year of follow-up, the patient has a satisfying urinary function, however, the sexual state was not assessable because of the persistent psychiatric disorder (Figure 3).

## Discussion

Penile self-mutilation is a rare traumatic urological emergency that requires prompt management. The case we report exemplifies the emergency challenging factors we faced, surgically, and psychologically. Klingsor syndrome is rare. It includes all genitalia self-mutilation in psychotic patients and also in religious delusions [5,6]. In male genitalia self-mutilation is 80% associated with psychosis [7]. The term «klingsor» refers to a character in Wagner´s germen opera who made a self-inflicted castration to have the privilege to earn a place with the prestigious brotherhood of knights of the holy grail [8]. The British Association of urological surgeons developed a consensus for the management of male genitalia emergencies in case of penile amputation. In their proposed algorithm, they suggested a psychiatric review and based the guidelines on whether the phallus is salvaged or not, to perform reimplantation using amputated phallus in a microsurgical specialist center or jointly with microsurgeons within 24 hours. In our case, the patient did not conserve the amputated phallus, reimplantation was impossible to perform. According to British association of urological surgeons (BAUS) guidelines, similar cases management depends on stump quality for voiding and sexual functions. They recommended a split-skin graft or primary closure of the skin in case penile stump was not available [9].

Rudy and Borden technique was first described for partial penile amputation in glan penis tumors [3]. A general or local anesthesia may be used. The cavernous bodies cut must be transversal and linear preserving the skin above and the spongious body and urethra below. Then, a careful hemostasis is done by ligating the dorsal pedicle using a slow resorption thread. The corporeal bodies albuginea is closed. The neomeatus is made by punching the dorsal skin flap previously preserved. The main technical issue we faced was the preservation of a significant skin flap owing to titled sloping cut, which constrained us to reduce the penile stump length by a 0.5cm cavernous bodies resection; doing so we kept 1cm of spongious body and urethra ahead that les us to perform a tension-free uretrocutaneous anastomosis. This technique has demonstrated that functional results depend on the length of the penile stump, especially on the quality of erections. A satisfying sexual function is possible when the penile stump length is at least 12cm. The main functional complication of this technique is the burial of penile stump that exposes to hygienic issues, infection, and alteration of voiding function. Our case brings light to the requirement of psychiatric management. In fact, our patient reoffends two years after his first self-harming behavior. There are some risk factors that were historically questioned, including but not limited to unresolved sexual conflict, absence of a confident and reliable mal figure, and masochistic behavior promoted by an overcontrolling mother [1,10].

## Conclusion

Many challenging factors are intervening in the urologic management of penile self-inflicted amputation, length of the penile stump, the preservation of the phallus amputated, and the quality of post-operative care among others. There are limited data describing the main surgical treatment in cases of the aforementioned requirements unavailability. The aim of this technique is to avoid perineostomy which compounds significantly the quality of life. It may be presumptuous to settle for the surgical approach only, psychiatric treatment features strongly in the overall management by treating the acute crisis, following the patient and his family circle during the urologic recovery process, and precluding recurrences.
